# Comparison of legislation, regulations and national health strategies for palliative care in seven European countries (Results from the Europall Research Group): a descriptive study

**DOI:** 10.1186/1472-6963-13-275

**Published:** 2013-07-17

**Authors:** Karen Van Beek, Kathrin Woitha, Nisar Ahmed, Johan Menten, Birgit Jaspers, Yvonne Engels, Sam H Ahmedzai, Kris Vissers, Jeroen Hasselaar

**Affiliations:** 1 Department of Radiation-Oncology and Palliative Medicine, University Hospital Gasthuisberg, Leuven, Belgium; 2 Department of Anesthesiology, Pain and Palliative Medicine, Radboud University Nijmegen Medical Centre, Nijmegen, The Netherlands; 3 Academic Unit of Supportive Care, School of Medicine and Biomedical Sciences, University of Sheffield, Sheffield, UK; 4 Department of Palliative Medicine, University of Bonn, Palliative Care Centre, Malteser Hospital Bonn/Rhein-Sieg, Bonn, Germany; 5 Department of Palliative Medicine, University of Goettingen, University Medical Clinic, Goettingen, Germany

**Keywords:** Palliative care, Government regulation, European Union, Health policy, Legislation, Health plan implementation

## Abstract

**Background:**

According to EU policy, anyone in need of palliative care should be able to have access to it. It is therefore important to investigate which palliative care topics are subject to legislation and regulations in Europe and how these are implemented in (national) health care plans. This paper aims to deliver a structured overview of the legislation, existing regulations and the different health care policies regarding palliative care in seven European countries.

**Methods:**

In 2008 an inventory of the organisation of palliative care was developed by the researchers of the Europall project. Included were two open questions about legislation, regulations, and health policy in palliative care. This questionnaire was completed using palliative care experts selected from Belgium, England, France, Germany, the Netherlands, Poland and Spain. Additionally, (grey) literature on palliative care health policy and regulations from the participating countries was collected to complete the inventory. Comparative analysis of country specific information was performed afterwards.

**Results:**

In all countries palliative care regulations and policies existed (either in laws, royal decrees, or national policies). An explicit right to palliative care was mentioned in the Belgium, French and German law. In addition, access to palliative care was mentioned by all countries, varying from explicit regulations to policy intentions in national plans. Also, all countries had a national policy on palliative care, although sometimes mainly related to national cancer plans. Differences existed in policy regarding palliative care leave, advance directives, national funding, palliative care training, research, opioids and the role of volunteers.

**Conclusions:**

Although all included European countries have policies on palliative care, countries largely differ in the presence of legislation and regulations on palliative care as well as the included topics. European healthcare policy recommendations should support palliative care access across Europe.

## Background

With the aging of the European population and an expected increase in mortality rates [1], a vision on palliative care development at a European level is increasingly important. The Council of Europe [2] and the WHO [3] formulated recommendations for the integration of palliative care into the national health care systems. They concluded that any person in need of palliative care should be able to access palliative care without undue delay, in a setting which is, as far as reasonably feasible, consistent with his or her needs and preferences and regardless of cultural, ethnic or other background. Public health policy must acknowledge people’s right to high-quality palliative care whatever the nature of the disease they suffer from and this should not depend on the financial abilities of patients or their informal caregivers. Because several European countries already have national initiatives regarding palliative care, it is relevant to consider to what extent European countries succeed to incorporate the advices from the Council of Europe and the WHO in their health care systems. This points to the importance of the development of quality indicators for palliative care [4] as several studies have attempted to define outcome or quality parameters in palliative care [5-7]. However, also the health system development in a certain region largely influences quality improvement in palliative care [8]. As part of an integral quality approach to palliative care, investigating (European) differences in health policy are pivotal, for example legislation and policy regulations. This paper therefore focuses on regulations, legislation and national health care strategies concerning palliative care in seven European countries to answer the following research questions:

1. What kinds of laws or other regulations (national/regional) regarding palliative care exist in the participating European countries?

2. Is there a national and/or regional health policy regarding palliative care and how is this implemented?

3. What palliative care topics are subject to health policy regulation and plans?

## Methods

The design and performance of this study comprised four phases. As a first step, in 2008, an inventory for the organization of palliative care was developed including a questionnaire with open questions. A careful process of agreement on definitions and terms, including the preparation of a concise glossary of terms [9] preceded the questionnaire to allow for the comparison of data between the different countries. The glossary and the questionnaire were completed during several project work conferences with all participating partners. In summer-fall of 2008 the partners of each participating country were asked to complete the inventory for his/her country, including two questions about legislation and national policy on palliative care.

Secondly, the partners contacted national palliative care experts in their country. In total, up to 6 to 12 key persons per country were consulted to further complete the questionnaire, taking into account the glossary of terms (professors in palliative care, professional care providers as well as policy makers/developers from palliative care organizations). Experts were approached using the network of the Europall research group.

Thirdly, (grey) literature was performed to further add to the information provided by the experts. This literature search focused on specific information per country because regulations and health policy are likely to be published in national or regional reports from official bodies (e.g. palliative care reports in each country, publications of palliative care organizations). In addition, experts handed over policy reports. Inclusion of grey literature was restricted to reports from government agencies or scientific research groups, white papers, or websites from national organizations and limited to the seven participating countries. Internet databases such as Medline, PubMed and Google scholar were searched for extra information about palliative care policies in the participating countries, using snowball sampling which has been considered fruitful for the inventory of complex policy interventions [5].

Fourthly, a comparative analysis of the completed surveys was performed. Because the analysis of answers to the sub-question on finances turned out to be a complex separate study field, it was decided not to include this in the analysis for this manuscript. Phases are summarized in Table 1.

**Table 1:** Palliative care inventory study design

*Phase I*	Development of the inventory			
	*-development of glossary of terms*[9]			
	*-development of questionnaire on organization of palliative care*			
	*> including two questions on legislation and regulation of palliative care in Europe*			
		1. What kinds of laws regarding palliative care do exist? (plus year established)		
		(Please specify: regional or national; name and summary of content. We do not mean laws about volunteers etc., although it might be linked to palliative care; laws about euthanasia etc. can be mentioned if they also mention palliative care)		
		2. Is there a national and/or regional health policy regarding palliative care in your country?		
			a. If yes, please describe (development, budget, aims)	
			b. Describe the implementation of the plan, regarding the organization of services	
			c. Is there a national / regional palliative care association/federation/organization in your country? Please describe on national and regional basis	
			d. How is palliative care financed? Please describe, also relevant budgets:	
			-Public /private/voluntary	
			-Is it dedicated for palliative care?	
			-Differences between care settings?	
*Phase II*	Consultation with 6 to 12 key persons per country to further complete the questionnaire,			
*Phase III*	(grey) Literature study			
	*-Collecting of palliative care policy documents per country*			
	*-Additional snowball sampling based on pubmed search, expert consultation, internet search.*			
*Phase IV*	Comparative analysis based upon the country specific information.			

For the involved countries, ethical approval was not required as this study was based on reviewing published and unpublished literature and consultation of experts.

## Results

### Palliative care regulations

**Germany** was the first country to have specific laws concerning palliative care; Social Code Book XI as well as V/§39a (introduced in 1997) cover regulations for palliative care [10]. In 1997, insured persons were entitled to allowances for inpatient or day care in hospices. In 2001, these regulations were extended to the delivery of outpatient palliative care by hospice services, including the services of hospice palliative care volunteers visiting the patients at their homes or elsewhere. Following *the Statutory Health Insurance Competition Strengthening Act (SHI-CSA) *[11] of April 2007, new paragraphs were inserted in the Social Code Book V, entitling patients, including children, to outpatient specialist palliative care when needed. The German guidelines for specialised palliative home care services (SAPV), developed by G-BA (Federal Joint Committee) came into force in March 2008. They include the aims of SAPV, requirements of the teams (qualification, multi-professionalism, organisation), content and extent of services, and quality assurance. SAPV teams and the delivery of care must fulfil detailed criteria in order to receive remuneration from statutory health insurers.

In **France**, law 99–477 of 9 June 1999 [12] was designed to ensure the right to adequate access to palliative care and supportive care for any citizen requiring this care. All hospital units and other healthcare institutions are obliged to provide pain and palliative treatment. Patients are allowed to refuse further investigations or treatment. Caregiver’s leave is stipulated for any ascendants, descendants or persons living in the home of a patient requiring palliative care (this measure has been applied only since 2010). The law 2005–370 of 22 April 2005 [13] (concerning patient rights and the end-of-life) allows, at the terminal or advanced phase of an incurable and serious disease, the possibility, after informing the patient or the patient’s representative (“person of confidence”), to relieve the patient’s suffering by a treatment which, as a side effect, can shorten the patient’s life. It authorises “advance directives” indicating the person’s wishes concerning his/her end of life and defining the conditions of limitation or discontinuation of treatment. The doctor is required to take these wishes into account when they have been expressed during the previous three years. The law also specifies that hospitals and other healthcare establishments must identify the departments in which palliative care is dispensed. In addition, every department needs to define how many employees have had specific training in palliative care and how many beds are identified as palliative care beds. A circular [14] published in March 2008 by the French Ministry of Health contain regulations for dedicated palliative care beds, mobile hospital support teams, palliative care units, home hospitalisation and palliative care networks. In the French system, which clearly distinguishes between hospital (predominantly public/quasi-public) and primary care (predominantly private), palliative care networks are important to maintain continuity between healthcare organisations and home care.

The **Belgian** law concerning palliative care, enacted in 2002 [15], declares that every citizen has the right to receive palliative care (and/or information about it), to receive information about his illness and warrants the accessibility to palliative care. The law defines palliative care as the total care provision for patients whose life threatening disease no longer responds to curative therapies. The major aim is to offer the patient and his/her next of kin as much quality of life as possible and a maximum of autonomy. For the support of these patients, multidisciplinary care on physical, psychiatric, social and moral level is considered pivotal. A broad commission has to report the implementation and the improvement of different palliative care deliveries to the government every second year, and annual progression reports are delivered by the Minister of Public Health and Social Affairs. The same year 2002 euthanasia was legalised in specific circumstances [16]. One precondition for the use of euthanasia is that the physician always needs to inform the patient about palliative care. Since 1993 many initiatives have been undertaken in **Belgium** at a federal and at community level to enable and support palliative care. There are Royal Decrees about palliative care, which can be considered as detailed guidelines for palliative care networks, hospital support teams and palliative home care teams. They stipulate what services are necessary, their geographical distribution, conditions and minimum criteria for staff, the content of their work, and sometimes funding of different palliative care services [17].

England, the Netherlands, Poland and Spain do not have specific palliative care laws, but palliative care is mentioned in general health care laws. In **Spain** the care for the chronically ill and terminally ill is mentioned in a law about the National Health System [18] and in **Poland** insured persons are entitled to palliative care in the law on Universal health insurance_._ In the **Netherlands** palliative care is considered a part of the regular health care legislation *(e.g. the Dutch Act of Agreement on Medical Treatment; WGBO) *[19]. A Euthanasia law exists (Termination of Life on Request and Assisted Suicide; published in 2002), stating amongst others that no reasonable alternative should be available for patients (although palliative care is not explicitly mentioned). In Poland there is a statement in the Code of Medical Ethics that obliges doctors to care for patients with incurable diseases and that offers patients the possibility to resign from intensive care at the end stage of incurable diseases. In **England** the Mental Capacity Act of 2005 [20] is an act of the Parliament of the United Kingdom which applies in England and Wales and came into force in April 2007. Its primary purpose is to provide a legal framework for acting and making decisions on behalf of adults who lack the capacity to make particular decisions for themselves. In **Poland**[21] there is a statement in the Code of Medical Ethics that obliges doctors to care for patients with incurable diseases and that offers patients the possibility to resign from intensive care at the end stage of incurable diseases.

In **Spain**, a Royal Decree (1030/2006) [22] determines the specific (not only palliative care) services to be provided in each health care sector as well as the mechanism for their modernisation and improvement. It defines the fundamental principles to guarantee the availability and equal access to palliative care at both the primary and secondary levels of care. An overview of the palliative care regulations is presented in Table 2.

**Table 2:** Legislation and other national/regional regulations about palliative care

	**Belgium**	**England (UK)**	**France**	**Germany**	**Netherlands**	**Poland**	**Spain**
**Laws regarding PC**	*Law 14 June2002* to ensure the right of access to palliative care	/	*Law 99–477 9 June1999* designed to ensure the right of access to palliative care	*National level, Social Code Book V* (introduced 20. December 1988) and *Social Code Book XI* (introduced 26 May 1994) § 39a covers in- and outpatient hospice services	/	/	/
				Amendment § 39a deals with special requirements for the care in children’s hospices			
				*The Statutory Health Insurance Competition Strengthening Act (SHI-CSA)*, April 2007: Incentives for better coordination of care			
			Law 2005–370 of 22 April 2005 concerning patient rights and the end of life				
**Other regulations concerning PC**	*Royal Decrees* concerning minimum service provision requirements in different PC settings and funding, free medical care for PC patients at home and palliative caregivers leave		*Circulars published in 2008 by the French Ministry of Health:* Healthcare organisation guidelines for palliative care services	2007, *GB-A*: new regulations on specialised palliative home care services	*Agreement palliative Terminal Care* Funding and regulations of specific PC settings	Point in Code of Medical Ethics: obligation for care for patient with incurable diseases and the possibility of resign from intensive care at the end stage of incurable diseases.	*1030/2006 Royal Decree, on September 15* defines the fundamental principles to guarantee the availability and equal access to PC

### National plans on palliative care

As palliative care and its provision is defined in the **Belgian** legislation [15] and several Royal Decrees act as healthcare organisation guidelines for the different palliative care services, there is no separate national palliative care policy. The National Cancer Plan [23] (March 2008) however states that the expansion of palliative care shall be actively supported. Palliative care has gained increased recognition within the policy arena in **the United Kingdom**. The so-called 1995 Calman-Hine Report was crucial in influencing plans for service development in cancer. Since the year 2000, several important national and regional initiatives have been launched to promote access to end-of-life care and to improve quality of care, including the NHS Cancer Plan of September 2000; an action plan to include palliative care in 34 regional cancer networks; the ‘NICE clinical guidance on supportive and palliative care for adults with cancer (2004)’ [24] The End of Life Care initiative (launched 2003) incorporating the Gold Standards Framework (GSF), the Liverpool Care Pathway (LCP) for the dying patient, and the Preferred Place of Care tool (PPC); The End of Life Care Programme [25] comprising a comprehensive framework aimed at improving high quality care across the country for adults in the last phase of life; and the initiative “Better care: Better Lives” improving outcomes and experiences for children, young people and their families living with life-limiting and life-threatening conditions” [26].

According to the palliative care law of 1999 [12], structures and organisation of palliative care and pain management must be described in the **French** regional health organisation plans (SROS). The SROS must determine the resources (such as mobile palliative care teams, palliative care units, home hospitalisation beds, palliative care networks) necessary to achieve the set objectives**.** In addition, palliative care was the subject of three governmental plans in France (1999–2001, 2002–2005 and 2008–2012). Following the Law of April 2005 [13], the Ministry of Health created a national surveillance committee on the development of palliative care and end of life supportive care in 2006. In 2008, this committee proposed a national policy for the development of palliative care, accompany the implementation and deployment of this policy, and evaluate application of legislative and regulatory texts concerning palliative care and end of life supportive care. A third Governmental plan [27] for the development of palliative care (2008–2012) was launched in 2008 for the further development of intra- and extramural palliative care, to pursue the development of training and research in palliative care and to improve the development of support and training for the paramedics.

In 2005, the **German** Bundestag officially declared the improvement of palliative care as a priority. The coalition treaty (CDU, CSU, and SPD) mentioned palliative care for the first time stating “that there is a particular need for improvement in the care and treatment of people in the final stages of their lives. Many people, even patients with serious illnesses, would like to be cared for at home to the very end, so the offered services should take this need into account. For this reason, the legal provisions governing the services, the contractual rights and obligations and the funding of statutory health and long-term care schemes must include rules designed to guarantee better palliative care” [28]. Health care reform was declared one of its top priorities for 2006 and in November 2005 they stipulated, among others, the promotion of incentives for better coordination of care, to raise efficiency and to improve quality of care [29]. As a result the entitlement to out-patient specialist palliative care was implemented in a law in the next electoral term (*Statutory Health Insurance Competition Strengthening Act (SHI-CSA)).*

For 1998–2003 the **Dutch** Minister of Health, Welfare and Sport initiated a stimulation programme. The underlying principle was that palliative care should be provided as much as possible by doctors, nurses, care workers and other care providers who work in regular non-private facilities. In this way access and availability to palliative care should be improved. The stimulation programme encompassed encouragement of research and innovative projects, promotion and guidance of palliative care and stimulation of the integration of hospice facilities. In 2007 the secretary of the Ministry of public health, welfare and sports did a proposal for a new national program 2007–2010 Palliative care Plan’ (Plan van Aanpak Palliatieve Zorg’ [30]) with three main topics: the organisation and finance of palliative care, the improvement of quality and transparency of palliative care and education and palliative care competencies (extra training).

There is no separate national palliative care plan as such in **Poland**. For the years 2005–2010 palliative care was included into the National Cancer Program, which allows the development of palliative care in-patient and out-patient services included into oncology units by financing support. Each oncology centre (usually situated in each capital of the province) should have a palliative care inpatient and outpatient unit or at least a hospital support team when formation of a separate palliative care unit is impossible. Although there is no national policy, the person responsible for the organisation of palliative care is the National Consultant of palliative medicine with regional consultants in each province (Voivodes). The National Consultant of palliative medicine (a physician with a specialty in palliative medicine) focuses on developing palliative care in each administrative district with access to at least an out-patient unit and home care. The **Spanish** regulations about palliative care have followed most principles and recommendations made by international organisations. This resulted in the National Palliative Care plan which was enacted in 2001 [31] and conceived to care for all patients in need of palliative care within the public sector. According to the vision of the plan, palliative care is to be nationwide available, on a free basis, and with no distinctions of territory, economic resources or accessed information [32]. The National Palliative Care Plan also regulates the use of opioids for symptom control in the incurably ill and pays attention to non-cancer patients. A regional palliative care plan exists, or is under development, in several autonomous communities (15 of the 17 autonomous communities have some type of regional palliative care plan). On March 17^th^ 2007 the Palliative Care Strategy of the National Health System, conceived as a tool for implementing the national plan and to support Autonomous Communities in the implementation of their regional programmes [33], was approved by the Inter-territorial council. The National Strategy seeks to reduce differences between regions in order to make palliative care thoroughly available within the national health system across the country. An overview of the palliative care policy plans can be found in Tables 2 and 3.

**Table 3:** Overview of the national and/ or regional health policy plans regarding palliative care

	**Belgium**	**England (UK)**	**France**	**Germany**	**Netherlands**	**Poland**	**Spain**
**National and/or regional health policy regarding palliative care**	*National Cancer Plan* (10-03-2008)	The NICE clinical guidance on supportive and palliative caresupport	Third Governmental plan 2008–2010: for further development of intra- and extramural palliative care.	Health Reform since 2006: promotion of modern forms of care such as integrated care and palliative care	2007-2010: ’Plan van Aanpak Palliatieve Zorg’: it consist of three topics:	There is no national palliative care plan.	*National Palliative Care Plan (2001)* promotes the design and implementation of services based on regional needs assessment, and encourages routinely adopting indicators and standards to assess progresses and care outcomes. It follows different strategic lines of action that are patients and families centred
		NHS Cancer Plan- Sep 2000-					
			- To pursue the development of formation in palliative care and of research in palliative care.				
		End of Life Care initiative in England-launched 2003					
			- Development of care for the carers				
		End of Life Care Strategy (July 2008)					
		National Strategy on Children’s Palliative Care launched 2008					
							A regional palliative care plan exists, or is under development, in several autonomous communities.
					- Organisation and finance of palliative care (optimal organisation and arrangement of tasks, products and service of palliative care organisations rural, regional and national)		
					- Improvement of quality and transparency of palliative care		
					- Education and assistance of competencies (extra training)		
						2005–2010: PC is included into the National Program of Cancer Treatment.	
**Implementation of the national palliative care plan**		-End of Life Care Strategy (July 2008) – England	-Implementation led by the government and carried out by the regional health care agencies (24 regions)		The Ministry composed a platform of organisations involved in palliative care	-The National Consultant of palliative medicine (attached to the Minister of Health)	−2007 march 17: The Palliative Care Strategy of the National Health System approved by the Interterritorial council
		-The Welsh Collaborative Care Pathway Project					
						-Regional consultants of palliative medicine in each province	
		-The Scottish Partnership for Palliative Care					
							−8 of the 17 autonomous communities have a regional palliative care plan
		-All-Ireland Institute for Hospice and Palliative Care					
**National Palliative Care organisation**	3 Federations of PC:	- NCPC (national council for PC England-Wales-Northern Ireland)	SFAP (Société d’Accompagnement et de soins palliatifs)	DHPV (Federal Hospice Working Group)	AGORA (Landelijk Ondersteunings-punt Palliatieve Zorg)	Polish Association for PC	SECPAL (Spanish Society for PC )
	FPZV (Flanders), FWSP (Walloon region), FBSP (Brussels)					Polish Association for Palliative Medicine	
				DGP (German Association for Palliative Medicine*)*			
		- The Scottish partnership for PC					


### Cross-national comparison

In this study covering seven European countries, the following topics appeared as subject to regulations and palliative care plans: definition of palliative care, rights to palliative care, access to palliative care, palliative care provision, quality assurance, patient allowances, palliative care leave, advanced directives, funding of services, palliative care research and training, and opioids availability. Three countries (Belgium, France and Germany) have specific laws on palliative care mostly to ensure the right of access to palliative care. An explicit right to palliative care was mentioned in the Belgium and in the French law. Access to palliative care and palliative care provision was mentioned by all countries, varying from explicit regulations to policy intentions in national plans. Also, all countries have a national policy on palliative care, although sometimes mainly related to national cancer plans (see Table 4). Of the seven investigated countries, five countries have specific national palliative care plans, although the content differs (see Table 5). Similarities in England, Germany, Netherlands and Spain are that they deal with quality assurance whereas research in palliative care is mentioned in the French, Dutch and Spanish policies. Differences relate to policy on access to and use of opioids. Variations in palliative care training for staff and volunteers as well as funding of palliative care also exist. In all countries there are regulations (either in laws, royal decrees, national policies) about patients’ financial contribution to palliative care (often almost free of costs). Other regulations exist on treatment relationship or planning and financing of palliative care services (Belgium and Spain), as does the ‘Agreement Palliative Terminal Care’ in the Netherlands. In Poland, the national consultant of Palliative Medicine guards the presence of at least one inpatient unit and home care in each administrative district. A palliative care leave was mentioned in three countries, Belgium, France, and Germany.

**Table 4:** Overview palliative care laws, regulations, national palliative care plans

	**Belgium**	**Netherlands**	**England**	**France**	**Poland**	**Spain**	**Germany**
**Specific PC laws**	*X*			*X*			*X*
**General health care laws with mentioning of PC**		*X*	*X*		*X*	*X*	
**Other regulations concerning PC**	*X*	*X*		*X*		*X*	*X*
**National PC plan**		*X*	*X*	*X*		*X*	*X*
**National cancer plan including PC**	*X*		*X*		*X*		

**Table 5:** Coverage of existing legislation, regulations and health care plans regarding palliative care

	**Belgium**	**England (UK)**	**France**	**Germany**	**Netherlands**	**Poland**	**Spain**
**Definition of PC mentioned**	A				D		
**Right to PC**	A		A	A			B, D
**Access to PC**	A	E	A	A, B, D	D	E	B, C, D
**PC provision**	A, B, E	E	A, B, D, E	A, B, D	B, D	E	B, D
**Quality assurance**	A	D		A, B, D	D		D
**Patient allowances**	B	C	C	D	C	C	C
**PC leave for informal caregivers**	B		A	B			
**Advance directives**	C	C	A		C	C	
**National funding PC services**	B, E		E	A, B, D	B, D	A	
**PC Training**		E	B, D	A, C	D		D
**Research**			D		D		D
**Opioids**			D				D
**PC volunteers**			D	A, B, C	C		

A. Palliative care laws.

B. Palliative care regulations.

C. General health care laws/ system.

D. National palliative care plans.

E. National cancer plan.

Additional note of the authors: after closure of our study, in May 2011, the Spanish government approved a law dedicated to palliative care and the right to a dignified death [34].

## Discussion

Although a thorough method was used to complete the inventory, it remains difficult to get a complete and actual overview of a whole country. In France, the United Kingdom, Poland, Germany and Spain, different regions may have developed regional palliative health care policies. In this article, this was solved by giving a general overview with some details for regions with specific regulations. As every country had their own appointed researcher, there are differences in the way the key persons were contacted, how many key persons responded and how the grey literature was consulted. As countries have different health care systems and different cultures conclusions should be interpreted carefully.

In the light of European recommendations [1,2] on palliative care it should be remarked that palliative care can be concluded a crucial part of health care at a policy level in all countries investigated. Considering that national palliative care programs are part of cancer care in some countries, the status of palliative care in non-cancer care needs further attention in policymaking. The access of patients to palliative care provisions is addressed in all national policies on palliative care. Palliative care training, research priorities, palliative care leave, advance directive procedures, and national funding, however, vary largely between countries and receive future attention. The regulations concerning opioids are explicitly addressed in two national care plans. This calls for future research as a recent ESMO/EAPC study concluded wide variation between (Eastern and Western) European countries in opioid availability [35]. Considering the wide variation in topics addressed in palliative care plans and regulations, it is recommended that a European quality indicator set for palliative care not only addresses outcome parameters but also health policy items. An advantage of such an approach will be that health policy indicators can measure to what extent appropriate preconditions for palliative care delivery to patients are available in a country. Such health policy indicators are relatively easy to establish if one has access to health policy evaluation documents in a certain country. This also offers the opportunity for so-called resource-poor countries to participate, which has been considered a challenge [36].

A European strategy to stimulate palliative care provision and policy is recommended, provided that differences in cultural and historical backgrounds are taken into account. A 2008 policy report on palliative care in Europe mentioned three possibilities to further develop palliative care: a) an approach in which further developments are considered the sole responsibility of national bodies, b) an approach in which the European Parliament formulates policy recommendations, c) an approach in which the European Union formulates legislation about palliative care [37]. Our study revealed important differences in palliative care policy at national levels at several points. The European Association for Palliative Care (EAPC) can have an important role in developing and comparing quality indicators for patients in the last phase of life to prepare (European) health policy recommendations for palliative care. This can be considered as first steps to reach consensus on what is good palliative care and how we can make sure that in Europe every patient in need of palliative care has adequate access to it.

## Conclusions

This study investigated and compared the legislation, regulations and national palliative care plans regarding palliative care in seven European countries.

European countries differ considerably in their policy on palliative care, partly due to cultural differences and historical influences. A right to palliative care is established in Belgium, France, and Germany, whereas all countries have policies on access to palliative care, palliative care provisions, and patient allowances. Differences exist in policies covering palliative care leave, advance directives, national funding, palliative care training, research, opioid regulations, and volunteering.

## Competing interests

The authors and co-authors have no competing interests.

## Author’s contributions

KVB: conception and design; acquisition of data; analysis and interpretation; drafting of manuscript; final approval. KW: conception and design; acquisition of data; analysis and interpretation; final approval. NA: conception and design; acquisition of data; analysis and interpretation; final approval. JM: conception and design; analysis and interpretation; critical revision of manuscript; final approval. BJ: conception and design; analysis and interpretation; final approval. YE: conception and design; critical revision of manuscript; final approval. SA: conception and design; final approval. KV: conception and design; critical revision of manuscript; final approval. JH: conception and design; drafting of manuscript; critical revision of manuscript; final approval. All authors read and approved the final manuscript.

## Pre-publication history

The pre-publication history for this paper can be accessed here:

http://www.biomedcentral.com/1472-6963/13/275/prepub
